# Comparative expression of the four enamel matrix protein genes, amelogenin, ameloblastin, enamelin and amelotin during amelogenesis in the lizard *Anolis carolinensis*

**DOI:** 10.1186/s13227-015-0024-4

**Published:** 2015-09-29

**Authors:** Barbara Gasse, Jean-Yves Sire

**Affiliations:** UMR7138, Institut de Biologie Paris-Seine (IBPS), UPMC Univ Paris 06, Sorbonne Universités, 75005 Paris, France; UMR7138, IBPS, CNRS, 75005 Paris, France

**Keywords:** Tooth, Enamel matrix protein, Gene expression, Evolution

## Abstract

**Background:**

In a recent study, we have demonstrated that amelotin (*AMTN*) gene structure and its expression during amelogenesis have changed during tetrapod evolution. Indeed, this gene is expressed throughout enamel matrix deposition and maturation in non-mammalian tetrapods, while in mammals its expression is restricted to the transition and maturation stages of amelogenesis. Previous studies of amelogenin (*AMEL*) gene expression in a lizard and a salamander have shown similar expression pattern to that in mammals, but to our knowledge there are no data regarding ameloblastin (*AMBN*) and enamelin (*ENAM*) expression in non-mammalian tetrapods. The present study aims to look at, and compare, the structure and expression of four enamel matrix protein genes, *AMEL*, *AMBN*, *ENAM* and *AMTN* during amelogenesis in the lizard *Anolis carolinensis.*

**Results:**

We provide the full-length cDNA sequence of *A. carolinensisAMEL* and *AMBN*, and show for the first time the expression of *ENAM* and *AMBN* in a non-mammalian species. During amelogenesis in *A. carolinensis*, *AMEL*, *AMBN* and *ENAM* expression in ameloblasts is similar to that described in mammals. It is noteworthy that *AMEL* and *AMBN* expression is also found in odontoblasts.

**Conclusions:**

Our findings indicate that *AMTN* is the only enamel matrix protein gene that is differentially expressed in ameloblasts between mammals and sauropsids. Changes in AMTN structure and expression could be the key to explain the structural differences between mammalian and reptilian enamel, i.e. prismatic versus non-prismatic.

## Background

Enamel is the highly mineralized tissue covering the tooth surface in tetrapods. In mammals, enamel is described as prismatic, i.e. with hydroxyapatite crystallites tightly packed into bundles in an organized pattern with complex orientations, whereas in non-mammalian tetrapods enamel is non-prismatic, i.e. generally with parallel crystals oriented perpendicular to the tooth surface [1]. The process of enamel formation, amelogenesis, has been extensively studied in mammals, and mostly in rodents [2, 3]. So far, the few studies of amelogenesis in non-mammalian tetrapods did not revealed important differences suggesting that the deposition of the enamel matrix occurred similarly in prismatic and non-prismatic enamel [4, 5]. Amelogenesis consists of two main stages: (1) the secretory stage, which consists in enamel matrix protein (EMP) deposition by cells of epithelial origin, the ameloblasts, then (2) the maturation stage, during which there is a progressive degradation of this matrix by ameloblast-secreted proteases, concomitant to an increased mineralization that represents more than 95 % of the enamel matrix at the end of this stage [6]. In mammals, the secretory ameloblasts are characterized by the presence of a cytoplasmic extension into the forming enamel matrix, the Tomes’ process [7]. In non-mammalian tetrapods, this cell extension does not exist, a feature which led the authors to consider that the presence of Tomes’ process was related to that of prisms [8]. However, it is still not clear whether this cell extension is directly responsible for prism formation or is induced by differences in the spatio-temporal expression of ameloblast-secreted proteins as recently suggested [9].

In rodents, six ameloblast-secreted proteins (ASPs) are expressed from early enamel matrix deposition to late maturation stage of amelogenesis. Among ASPs, three EMPs, amelogenin (AMEL), ameloblastin (AMBN) and enamelin (ENAM) are expressed from the beginning of amelogenesis until the onset of the maturation stage. AMEL, AMBN and ENAM are involved either in enamel matrix formation or in the control of the mineralization process, or in both, with AMEL representing circa 90 % of the forming enamel matrix [10]. These EMPs have important functions as demonstrated by the enamel disorders that occur when encoding genes are invalidated in mice [11, 12] and by the numerous mutations in humans leading to amelogenesis imperfecta [13, 14]. A fourth EMP, amelotin (AMTN) has been described in rodents, in which it is expressed during enamel maturation [15, 16]. The protein localizes to the basal lamina between the ameloblasts and the enamel surface [16, 17], where it is believed to be involved in the formation of the final, thin, non-prismatic enamel layer [18, 19]. In rodents, the two other ASPs, odontogenic ameloblast-associated protein (ODAM) and secretory calcium-binding phosphoprotein proline and glutamine rich 1 (SCPPPQ1) are not EMPs and their role is not well known [20]. ODAM expression pattern is similar to that of AMTN but SCPPPQ1 is expressed later during the late maturation stage [21, 22]. In addition, *ODAM* has not been identified in any sauropsid genomes so far [23].

The six ASPs belong to the secretory calcium-binding phosphoprotein (SCPP) family, whose genes arose through tandem duplications from an ancestral gene [24, 25]. Recently, all ASP encoding genes were found in the coelacanth genome [23], a finding that indicates their probable presence in the genome of the last common ancestor of sarcopterygians, and that supports the origin of the ancestral ASP gene earlier in the vertebrate history, probably around 530 million years ago (Mya) [26, 27].

For years, our research group has been interested in the story of SCPPs, more especially in their origin and relationships, and in the relation between changes in gene structure and putative modifications of protein functions [9, 28]. However, data on EMP expression during amelogenesis mostly accumulated in mammals, and this large amount of information contrasts with the poor knowledge of EMP expression during enamel formation in non-mammalian tetrapods [4, 29]. We have recently shown that *AMTN* was differently expressed in non-mammalian tetrapods when compared with the mouse. In the latter *AMTN* is expressed late during amelogenesis while in the lizard *A. carolinensis* and in the salamander *Pleurodeles waltl*, *AMTN* is expressed earlier, from the secretion stage onwards [9]. For a still unknown reason, the expression pattern and structure of *AMTN* drastically changed early in the mammalian lineage after its divergence from the sauropsid lineage [9].

In the present study, we wanted to check, in a sauropsid model organism, the dactyloid lizard *A. carolinensis*, whether or not the structure and the spatio-temporal expression of *AMEL*, *ENAM* and *AMBN* were different from those described in the mouse, as observed for *AMTN*. Previous studies have shown a similar expression pattern of *AMEL* in mouse and in two non-mammalian tetrapods, the scincid lizard *Chalcides viridanus* [30] and the salamander *P. waltl* [5]. However, on the one hand, scincids and dactyloids (a family close to iguanids) are largely evolutionary distant (>180 million years)—which explains why we included *AMEL* in our study—and, on the other hand, there were no data on the expression pattern of *ENAM* and *AMBN* during amelogenesis in non-mammalian tetrapods, although their gene structure was known in some non-mammals [28, 31, 32].

## Methods

### Ethics statement

All animal experiments conformed to the directives of the European parliament and of the council of 22 September 2010 on the protection of animals used for scientific purposes (Directive 2010/63/EU) and the French Rural Code (Article R214-87 to R214-137, Decree no. 2013-118 of 1st February 2013). Certificate of authorization for vertebrate animal experiment no. 75-600.

### Biological material

*Anolis carolinensis* specimens were a gift from the pet shop “La Ferme tropicale” in Paris. Several juvenile specimens were sacrificed and their lower and upper jaws were dissected. One sample was used for RNA extraction, and the other jaws were divided into two quadrants and fixed for in situ hybridization experiments.

### mRNA sequences

The *AMEL* and *AMBN* mRNA sequences used in our study were available in GenBank either as published mRNA or as computer-predicted (XM) sequences from sequenced genomes. They were:

AMEL: human, *Homo sapiens* [GenBank: NM_182680.1]; mouse, *Mus musculus* [NM_001081978.2]; opossum, *Monodelphis domestica* [XM_003341802.2]; crocodile, *Paleosuchus palpebrosus* [AF095568.1]; lizard, *A. carolinensis* [XM_008122480.1]; frog, *Xenopus tropicalis* [NM_001113681.1]; salamander, *P. waltl* [JX508595.1].

AMBN: *H. sapiens* [NM_016519]; *M. musculus* [NM_009664]; *M. domestica* [XM_007495519.1]; *Caiman crocodilus* [AY043290]; *A.carolinensis* [XM_008103732]; *X. laevis* [NM_001090020.1].

### RNA extraction, PCR and probe synthesis

Immediately after dissection the sample was immersed in liquid nitrogen and reduced to powder. Total RNAs were extracted and purified using RNeasy fibrous tissue Mini kit (Qiagen, France). RNAs were converted into cDNA by RT-PCR (RevertAid™ H Minus First Strand cDNA Synthesis Kit; Fermentas, France) using oligo(dT)18 primers.

Rapid amplification of cDNA end (RACE)-PCR was performed to recover the full length (including 5′ and 3′ ends) of *AMEL* and *AMBN* mRNA using SMARTer™ RACE cDNA Amplification Kit and Advantage 2 PCR Kit (Clontech) as previously described [28]. Primers were designed from the computer-predicted genomic sequences of these two genes in *A. carolinensis* using Primer3 v.0.4.0 [33].

5′RACE: AMEL-GSP1 (gene specific primer) antisense 5′-CATTGGGTGTTCTCCTGCATGTGGT-3′; AMEL-NGSP1 (nested gene specific primer) antisense 5′-GTGTGGGTTCAGTGCTGGATGTGGT-3′; AMBN-GSP1 antisense 5′-TGAATGGCATACCGTGGAATCTGGAC-3′; AMBN-NGSP1 antisense 5′-TGCAAACTGAATGGGCGTTTGCAGAGAC-3′.

3′RACE: AMEL-GSP2 sense 5′-GATGCCCCAGTTTCAACCAGCTCAT-3′; AMEL-NGSP2 sense 5′-CTCTTGAATCATGGCCACCAGCTGA-3′; AMBN-GSP2 sense 5′-ACTCAGGGCCCTTTCCTTCCTTTGGAT-3′; AMBN-NGSP2 sense 5′-AATGTGGGAAATGAGGCTGGTCTGG-3′.

For probe synthesis, specific primers were designed from the cDNA sequences of the four EMPs:

*AMEL*: sense 5′-TTTGCTATTCCATTGCCACA-3′; antisense 5′-GGCCATGATTCAAGAGGTGT-3′;

*AMBN*: sense 5′-ATGTTCTGCTCTGCCGCTAT-3′; antisense 5′-GCAGCTCCTTGGTTTGCTAC-3′;

*ENAM*: sense 5′-CAGCCTACATTTCCCCTTCA-3′; antisense 5′-CTGTGCCACTCCATTTCCTT-3′;

*AMTN*: see [9].

cDNA was amplified by PCR (GoTaq polymerase, Promega, France), inserted into a pCRII-TOPO vector containing T7 and SP6 promoters for in vitro RNA transcription (TOPO-TA cloning kit; Invitrogen, France), and transformed into competent *E. coli* TOP10F’ bacteria. Plasmids were purified (QIAprep Spin MiniPrep Kit; Quiagen, France) and linearized by PCR using M13 universal primers. Antisense RNA probes were synthesized using SP6 and T7 RNA polymerases (Riboprobe Combination System SP6/T7; Promega, France) in the presence of digoxigenin-UTP (Roche, France) and purified (ProbeQuant G-50 micro columns; GE Healthcare, France).

Sequence alignments were performed using the SeaView 4.3.3 software [34].

### In situ hybridization on sections

Samples were fixed overnight at 4 °C in Formoy’s solution (30 % formaldehyde 37, 10 % acetic acid and 60 % ethanol), and demineralized in 10 % acetic acid for 1 month at room temperature under gentle agitation. Samples were then dehydrated in ethanol, shortly immersed in toluene and embedded in Paraplast (Sigma, France). Eight µm-thick sections were obtained with a Leica RM2245 microtome, deposited on Superfrost PLUS slides (Fisher Scientific, France) and dried. They were then dewaxed in toluene, rehydrated through a decreasing series of ethanol then in PBS, treated with proteinase *K* (0.6 µg/ml) for 5 min at 37 °C, rinsed in PBS, post-fixed for 30 min in 4 % paraformaldehyde, rinsed again in PBS and then in 2X SCC. The slides were incubated overnight, at 65 °C, with the digoxigenin-labeled antisense probe (dilution: 1/150) in the hybridization buffer (50 % formamide, 10 % dextran sulfate, 1X salt, 1X Denhardt, yeast RNA 1 mg/ml). The following day, the slides were washed three times, at 65 °C, in the washing buffer (50 % formamide, 1X SCC, 0.1 % Tween 20), and rinsed, at room temperature, in the Maleic Acid Buffer Tween (MABT), pH 7.5. Non-specific binding sites were blocked for 2 h in a blocking solution (2 % blocking reagent, 20 % goat serum in MABT). Then, the slides were incubated overnight with the anti-digoxigenin antibody coupled to alkaline phosphatase (dilution: 1/1000) in the blocking solution. The next day, the slides were rinsed four times in MABT, then in NTM (NaCl, TrisHCl, MgCl_2_) buffer pH 9.5. The digoxigenin-labeled probes were revealed at 37 °C using NBT/BCIP (nitro blue tetrazolium chloride/5-bromo-4-chloro-3-indolyl phosphate). The slides were mounted in Aquatex mounting medium (Merck, France), and photographed (Olympus BX61 microscope).

## Results

### EMP gene structure in *A. carolinensis* and representative tetrapods

The full-length cDNA sequence of *ENAM* and *AMTN*, and the comparison of the gene structure with other tetrapod sequences were previously published [9, 28].

**Fig. 1:**
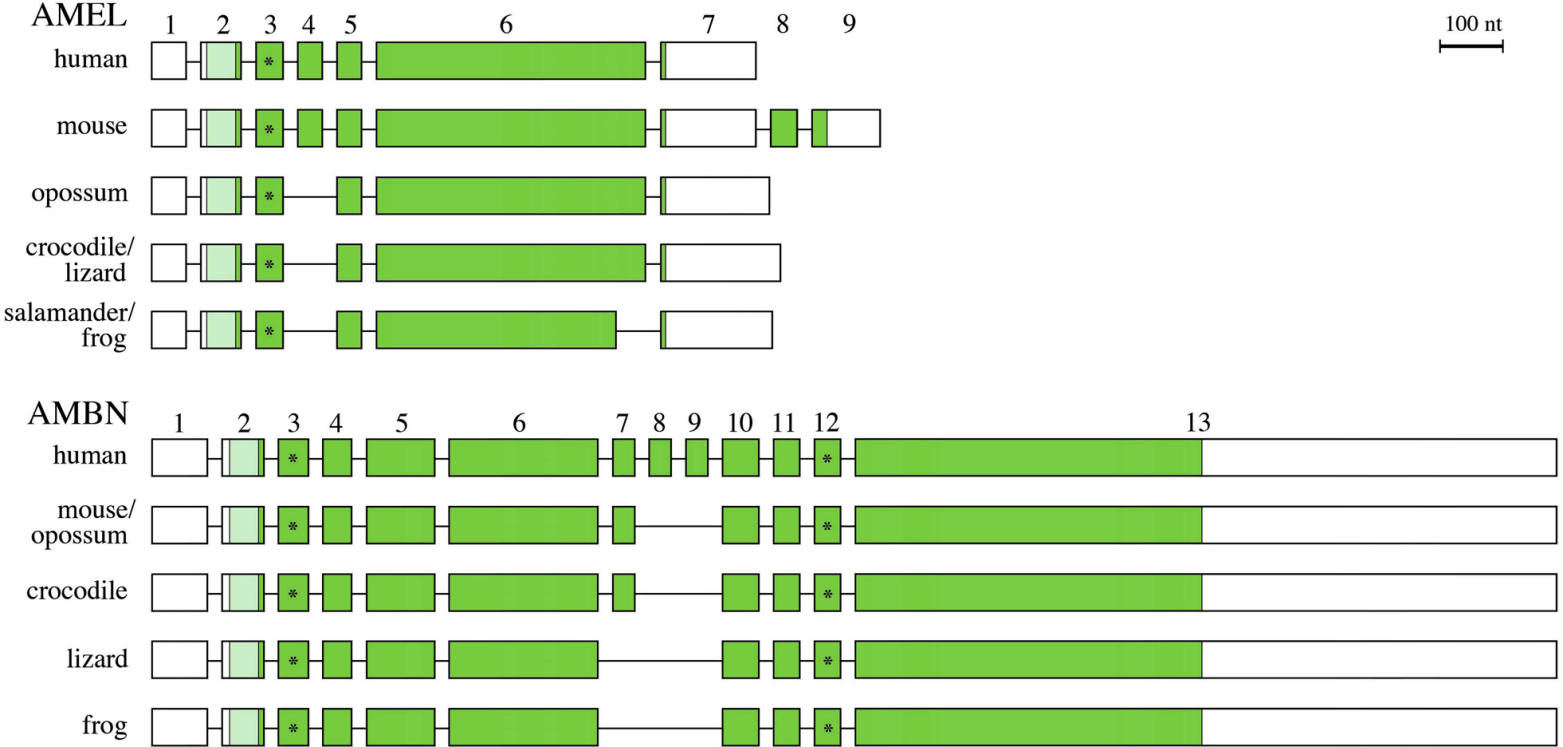
Structure of *AMEL* and *AMBN* in *A. carolinensis* and in representatives of main tetrapod lineages. Exons are represented by *boxes* and introns by *lines* (not at scale). Coding regions are in *dark green*; signal peptide in *light* *green*; non-coding regions in *white*. *Asterisk* potential phosphorylated Ser residue

*AMEL* and *AMBN* mRNA sequences of *A. carolinensis* were available in GenBank as computer-predicted sequences from the sequenced genome. RACE-PCR provided full-length sequence of these two transcripts. No alternative splice variant was found for either gene in our PCR products. The gene structure was defined using cDNA/gDNA comparisons and sequences were aligned to representative tetrapod sequences (Fig. 1).

The full-length sequence of *AMEL* transcript in *A. carolinensis* comprises 837 base pairs (bp), including UTRs. The computer-predicted coding sequence and the 3′UTR were confirmed while exon 1 sequence (60 bp) included in the 5′UTR (77 bp) was different from the predicted sequence. In this dactyloid lizard the *AMEL* structure is similar to that previously reported in tetrapods. The transcript is composed of six exons. It lacks exon 4, which is only found in placental *AMEL* and belongs to a minor transcript. *AMEL* cDNA sequence of *A. carolinensis* has been deposited in GenBank [accession No. KP792754].

The *AMBN* transcript of *A. carolinensis* is 1787 bp long, including UTRs. The 5′UTR consists of exon 1 (118 bp) and the beginning of exon two (32 bp). The gene structure is similar to other tetrapod *AMBN* sequences with the exception of exon 7 that is lacking as in frog *AMBN*. The computer-predicted mRNA sequence was quite different from the expressed transcript in (1) being 272 bp shorter, (2) starting the coding sequence with the ATG in exon three, and (3) lacking exon 4 and 6 bp at the end of the 3′UTR. *AMBN* cDNA sequence of *A. carolinensis* has been deposited in GenBank (No. KP792753).

### In situ hybridization of EMP genes during amelogenesis

**Fig. 2:**
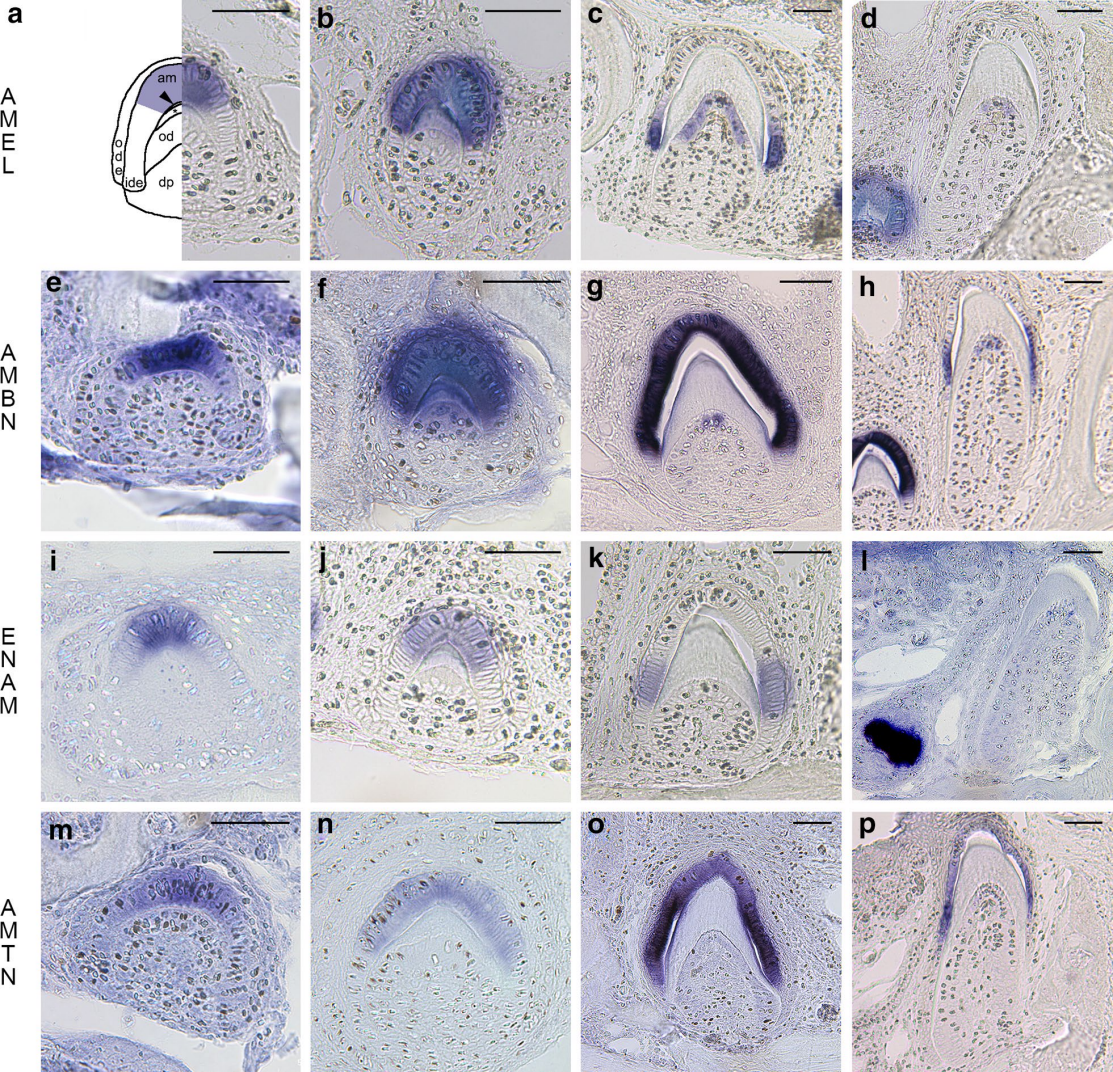
In situ hybridization of *AMEL, AMBN*, *ENAM* and *AMTN* during amelogenesis in *A. carolinensis*. Early secretory stage (**a**, **e**, **i**, **m**), secretory stage (**b**, **f**, **j**, **n**), early maturation stage (**c**, **g**, **k**, **o**) and late maturation stage (**d**, **h**, **l**, **p**). *A* interpretative drawing indicating the various cell populations shown in the pictures, *am* ameloblasts, *dp* dental papilla, *ide* inner dental epithelium, *od* odontoblasts, *ode* outer dental epithelium, *Asterisk* dentin, *arrowhead* enamel. *Scale bars* 50 µm

The expression of the four EMP genes was monitored in replacement teeth of *A. carolinensis* during four stages of amelogenesis: (1) early enamel matrix deposition, when a thin layer of predentin was deposited by the odontoblasts, a population of cells, ectomesenchymal in origin, responsible for dentin formation, and when ameloblasts were already differentiated around the tooth tip (Fig. 2a, e, i, m); (2) enamel matrix deposition and mineralization, when ameloblasts are active along the upper part of the teeth (Fig. 2b, f, j, n); (3) enamel matrix maturation, when mineralization increases from the tooth tip towards the tooth base, a stage during which enamel proteins are degraded by proteases (Fig. 2c, g, k, o); and (4) when most enamel is matured, tooth nearly to become functional and a new replacement tooth already formed (Fig. 2d, h, l, p). Although data on *AMTN* expression were published elsewhere, some original pictures were added on the figure for convenience of comparison (see [9] for a detailed description).

### Amelogenin

*AMEL* expression is first detected in secretory ameloblasts located at the tooth tip during early enamel matrix deposition (Fig. 2a), then transcripts are identified in the whole ameloblast layer facing the recently deposited enamel matrix (Fig. 2b). During the maturation stage *AMEL* expression is no longer seen in the ameloblasts at the tooth tip but is still present in those towards the tooth base, on which enamel matrix is still deposited. At this stage, *AMEL* transcripts are also detected in the odontoblasts facing the recently deposited predentin matrix (Fig. 2c). When the tooth is nearly functional *AMEL* transcripts are no longer detected in the reduced ameloblasts, but are still faintly present in odontoblasts in the upper region of the pulp cavity (Fig. 2d).

### Ameloblastin

*AMBN* expression is detected in the ameloblasts during early enamel matrix deposition (Fig. 2e). As matrix deposition progresses, *AMBN* transcripts are strongly labelled in the well-polarized ameloblasts (Fig. 2f). At the maturation stage, *AMBN* mRNA are strongly labelled in ameloblasts facing both the maturing (tooth tip) and the mineralizing (towards the tooth base) enamel matrix. Expression is also identified in a few odontoblasts located in the upper region of the pulp cavity (Fig. 2g). During late maturation stage, prior to tooth eruption *AMBN* is still detected in the ameloblasts near the dentin-enamel junction where enamel maturation is not completed, while it is no longer detected in the ameloblasts facing mature enamel. *AMBN* transcripts are still detected in a few odontoblasts (Fig. 2h).

### Enamelin

*ENAM* transcripts are detected early during tooth development, in ameloblasts at the early secretory stage (Fig. 2i). During enamel matrix deposition, *ENAM* expression is localized in the ameloblasts around the tooth tip (Fig. 2j). Later, *ENAM* mRNA are no longer labelled in the ameloblasts facing the maturing enamel at the tooth tip, while still present in the ameloblasts facing the immature enamel towards the tooth base, near the dentin-enamel junction (Fig. 2k). At the late maturation stage, *ENAM* expression was no longer identified in the reduced ameloblasts, including those located towards the tooth base (Fig. 2l). *ENAM* transcripts were never detected in odontoblasts or other cells of the dental organ.

## Discussion

In the present study, we provided (1) the full-length cDNA sequences of *AMEL* and *AMBN* in the lizard *A. carolinensis*, (2) the first description of *AMBN* and *ENAM* expression during amelogenesis in a non-mammalian species, and (3) the first description of *AMEL* expression in a dactyloid squamate. Together with previous data on the *ENAM* and *AMTN* gene structure obtained in our research group in this species [9, 28] our new sequence data allowed accurate comparison of the four EMP cDNA sequences in the main tetrapod lineages. Also, combined with recently published data obtained on *AMTN* expression our new results allowed to compare the four EMP gene expressions in the same lizard species one to another, and with published data in the mouse.

### Conservation and variations of tetrapod EMP gene structure

Sequencing mRNA allowed us to clarify the gene structure of *AMEL* and *AMBN* in *A. carolinensis*, especially in the 5′ region, including the UTRs. This was quite expected because these non-coding regions are highly variable and are difficult to find using computer-prediction, as they are not always conserved through evolution. Our results also confirm previous computer-predicted coding regions.

The 5′UTR of *AMEL* was different from the two predicted sequences in GenBank while the 3′UTR is identical. The structure of the coding sequence, composed of five exons (2, 3, 5, 6 and 7), is similar to that previously reported in other squamates, the iguanid *Iguana iguana* [35] and *C. viridanus* [30], in the amphibians *Rana pipiens* [36] and *P. waltl* [5] and in the coelacanth [23]. Recently, an additional exon was found between *AMEL* exons 5 and 6 in the iguanid lizard *Ctenosaura similis* [37]. This exon was not found in the *AMEL* transcripts of the closest species, *I. iguana*, which indicates independent exon recruitment in the *Ctenosaura* lineage (>30 Mya [38]). *AMEL* structure in reptiles differs, however, from that in mammals in lacking exon 4, which confirms the origin of this exon in mammals [39]. Nevertheless, in mice, exon 4 does not belong to the major *AMEL* transcript, and its role is not well understood to date. Moreover, lizard *AMEL* does not exhibit various splicing variants as described in the mouse, with additional exons 8 and 9 encoded in minor isoforms [39].

In GenBank, the computer-predicted *AMBN* sequence of *A. carolinensis* was not complete. The full-length sequence of *AMBN* mRNA, including both 5′ and 3′UTR, indicates the presence of ten exons, a gene structure similar to the one predicted by Kawasaki and Amemiya [23] in this lizard and to that of the frog sequence [32]. The *AMBN* structure of lizard and frog differs from that described in the crocodile *C. crocodilus* [31] and in mammals [40] in lacking exon 7. This finding suggests that exon 7 probably appeared in an ancestral amniote then was lost in the squamate lineage. In mammals, exon 7 sequence is variable and the encoded peptide does not contain important residues or motifs [40]. Moreover, exon 7 has duplicated several times, and independently, in some mammalian species [41, 42]. Although alternative splicing was reported in rat, mouse, human and pig *AMBN* [41, 43–45], only a single lizard transcript was found in the PCR products.

The *ENAM* structure in *A. carolinensis* was previously reported in the course of an evolutionary analysis of ENAM in tetrapods [28]. The main differences deduced from the comparison with mammalian *ENAM* structure are the absence of exon 3 and the presence of the additional exon 8b in the lizard sequence. In mammals, the former houses a putative, correct translation initiation site (ATG) suggesting the presence of two alternative (either long or short) signal peptides, the short signal peptide being ancestral. Exon 8b is also found in crocodile and marsupial *ENAM*, but absent in frog and lost in placental mammals. An RGD motif corresponding to a cell attachment sequence is present in the C-terminal region of all non-mammalian tetrapod ENAM. This motif is only present in some mammalian species, a finding which suggests limited functional constraints.

The *AMTN* structure has been recently studied in tetrapods, including in *A. carolinensis* [9]. In sauropsids and amphibians the *AMTN* structure greatly differs from that described in mammals. Compared to the mouse, lizard *AMTN* displays three additional exons, ends with a large exon 8 that encodes a RGD motif (shorter exon 8 and no RGD in the mouse) and does not possess exon 9 found in all mammalian sequences [9, 46].

Taken together, our comparative analyses of the four EMP gene structure in *A. carolinensis* versus mammals, including the mouse indicates that the *AMEL*, *AMBN* and *ENAM* structure are roughly similar in both lizard and mice, in contrast with the drastically different structure of *AMTN* [9].

### Similarities and differences in EMP mRNA expression during amelogenesis in lizard and mouse

In *A. carolinensis* during amelogenesis, the expression patterns of *AMEL*, *AMBN*, *ENAM* are spatio-temporally similar to those described in mammals, i.e. *AMEL* and *ENAM* are predominantly expressed in ameloblasts during the secretory and transition stages of amelogenesis, while *AMBN* displays a broader distribution from secretory to late maturation stages, its expression being maintained the longest of the three [18]. In contrast to what was previously described during amelogenesis in rodents [15], in lizard *AMTN* expression starts nearly simultaneously with the three other EMP genes, in secretory stage ameloblasts and goes on until late maturation stages, i.e. beyond the expression of the three other EMP genes including *AMBN*. This difference in spatio-temporal expression of *AMTN* could be explained as important changes in the function of the protein during evolution, from a wide expression pattern throughout amelogenesis in non-mammals to more restricted pattern in mammals [9]. These changes in *AMTN* expression could be the consequence of modifications in the regulatory region of the gene in an ancestral mammal [47]. However, to our knowledge there are no studies of the cis-regulatory elements in the EMP genes demonstrating their role in controling EMP gene expression during amelogenesis. Cis-regulatory elements were identified for *AMEL* [48], *AMBN* [49, 50], *AMTN* [51] and *ENAM* [52, 53] as well as some transcription factors e.g. Cbfa1 for *AMBN* [54]. However it remains unclear what could trigger expression of these genes during amelogenesis.

In addition, during tooth formation in *A. carolinensis*, the expression of two EMP genes *AMEL* and *AMBN* was also detected in the odontoblasts, although these cells are involved in dentin formation.

In a previous study of *AMEL* expression during amelogenesis in a scincid lizard, *C. viridanus* transcripts were detected in ameloblasts, from the secretory stage until the onset of the maturation process, but not in odontoblasts [30]. Different expression patterns of *AMEL* in these two lizards showing similar amelogenesis are quite surprising but could be related to the large evolutionary distance that separates the two species. Indeed, scincid and dactyloid lineages have diverged around 166 Mya [55], a period long enough for some changes to occur in *A. carolinensis* amelogenesis. In the latter the onset of *AMEL* expression in odontoblasts matches the onset of enamel maturation stage, and these cells remain labelled after *AMEL* expression has stopped in late maturation stage ameloblasts. More investigations are however needed to understand the reasons why *AMEL* is expressed by these odontoblasts. How is activated *AMEL*? Are the transcripts translated and the protein secreted? Does it play a role in reinforcing the dentin-enamel junction? In mammals, several studies have detected *AMEL* expression in pre-odontoblasts and in recently differentiated odontoblasts but never in later stages [56, 57]. The short distance between ameloblasts and odontoblasts at the onset of *AMEL* expression in mammals compared to the large distance between these cells in lizard, as they are separated by the dentine and enamel layers, could mean that the process leading to *AMEL* activation are different in lizards and mice.

In *A. carolinensis*, *AMBN* transcripts are detected concomitantly with the *AMEL* ones in the odontoblasts located at the upper region of the pulp cavity. This suggests that the two genes are activated simultaneously, probably by means of the same process. We have also to keep in mind that *AMEL* and *AMBN* are phylogenetically related genes [26]. In mammals, *AMBN* expression in odontoblasts has also been reported but only during early stages of amelogenesis [58, 59]. This again indicates that both genes probably have relationships.

## Conclusion

Our study reveals that among the four EMP genes *AMTN* is the only gene that displays major differences both in structure and in expression pattern during amelogenesis in lizard versus mice. The slight variations observed in the structure of the three other EMP genes seemed to have no consequence in their spatio-temporal expression during amelogenesis. In the amphibian, *Pleurodeles watlt* the expression pattern of *AMTN* during amelogenesis was similar to that described in the lizard [9]. A recent study in the mouse suggested that *AMTN* is involved in the establishment of the non-prismatic surface enamel layer and promotes calcium phosphate mineralization [19]. In non-mammalian tetrapods, enamel is not prismatic while it is prismatic in mammals. Nevertheless, the final surface layer that forms concomitantly to *AMTN* expression in the facing ameloblasts is prismless. Given the important role played by the enamel matrix proteins in the high organization of the enamel structure and its mineralization, and given that our comparison of the four lizard and mouse EMP genes pointed to only important changes in *AMTN* structure and expression, we conclude that these *AMTN* changes could be related to the transition between the non-prismatic enamel in non-mammalian tetrapods to the prismatic enamel in mammals.

## Abbreviations

AMBN: ameloblastin
AMEL: amelogenin
AMTN: amelotin
ASP: ameloblast-secreted protein
bp: base pairs
EMP: enamel matrix protein
ENAM: enamelin
GSP: gene specific primer
MABT: maleic acid buffer tween
Mya: million years ago
NBT/BCIP: nitro blue tetrazolium chloride/5-bromo-4-chloro-3-indolyl phosphate
NGSP: nested gene specific primer
No: number
NTM: NaCL, TrisHCl, MgCl_2_
ODAM: odontogenic ameloblast-associated protein
PCR: polymerase chain reaction
RACE: rapid amplification of cDNA end
RGD: arg-gly-asp
RT-PCR: reverse transcription polymerase chain reaction
SCPP: secretory calcium-binding phosphoprotein
SCPPPQ1: secretory calcium-binding phosphoprotein proline and glutamine rich 1
UTR: untranslated region
